# Upregulation of long noncoding RNA linc02544 and its association with overall survival rate and the influence on cell proliferation and migration in lung squamous cell carcinoma

**DOI:** 10.1007/s12672-022-00501-5

**Published:** 2022-05-30

**Authors:** Wei Wei, Teng Xu, Ying Zhang, Yong Huang, Xiang Wang

**Affiliations:** 1 Department of Cardiothoracic Surgery, Jinling Hospital, Nanjing University School of Medicine, Jiangsu, 210002 China; 2 Department of General Surgery, The Affiliated Hospital of Xuzhou Medical University, Jiangsu, 221000 China; 3 Department of Pathology, Jinling Hospital, Nanjing University School of Medicine, Nanjing, 210002 Jiangsu China; 4 Department of Medical Oncology, Cancer Center of Jinling Hospital, No. 34, 34 Biao, Yanggongjing Street, Nanjing, 210002 Jiangsu China; 5 Department of Medical Oncology, Xuzhou Central Hospital, 199 Jiefang South Road, Xuzhou, 221009 Jiangsu China

**Keywords:** linc02544, Lung squamous cell carcinoma, miR-138-5p, E2F3, Prognosis

## Abstract

**Background:**

Long noncoding RNAs (lncRNAs) exert crucial biological functions by regulating miRNAs, which are implicated in cancer progression and tumorigenesis. A previous study has indicated that lncRNA linc02544 expression is upregulated in lung adenocarcinoma, whereas, the role of linc02544 in LUSC is elusive.

**Methods:**

The differential linc02544 expression in LUSC tissues and adjacent non-tumor tissues were evaluated with RT-qPCR. Kaplan-Meier curve was conducted to evaluate the clinical prognostic significance of linc02544. Then cellular experiments were performed to assess the influence of linc02544 in LUSC proliferation, invasion, and migration, and a western blot assay was used to measure the metastasis-related protein levels. The downstream miRNAs were verified using the LncBase Experimental v.2 database and dual-luciferase reporter assay.

**Results:**

Linc02544 was overexpressed in LUSC tissues from positive lymph node metastasis-positive and TNM high-stage patients. Low linc02544 expression was associated with a longer survival rate. Downregulation of linc02544 by si-linc02544 restrained cell growth capacities, migration, and invasion abilities. Expression of MMP-2, MMP-9, and vimentin was decreased while E-cadherin was increased in si-linc02544 cells compared with that in untreated cells. Mechanistically, we identified that linc02544 acted as a sponge of miR-138-5p, which expression had a negative correlation. E2F3 was a potential target of miR-138-5p,

**Conclusions:**

Notably, high linc02544 expression was associated with severe clinical parameters and was a putative prognostic predictor for patients with LUSC. Downregulation of linc02544 may weaken the LUSC cell proliferation, migration, and invasion by regulating miR-138-5p/E2F3, which maybe serve as a biomarker for the prognosis and target treatment of LUSC.

**Supplementary information:**

The online version contains supplementary material available at 10.1007/s12672-022-00501-5.

## Introduction

As one of the fastest-growing malignant tumors, lung cancer has the highest morbidity and mortality worldwide [1]. The incidence and mortality of lung cancer have always been the first in China, posing a huge threat to the health and life of people and causing a huge social and economic burden [2]. Non-small cell lung cancer (NSCLC) accounts for about 85% of the total number of lung cancers [3]. In terms of personalized treatment, current research on NSCLC has been in-depth to the gene level [4]. Lung squamous cell carcinoma (LUSC) is one of the primary subtypes of NSCLC with high mortality. With the refinement of NSCLC treatment and the continuous development of molecular targeted therapy, novel tumor molecular markers related to the prognosis still needs to be updated to meet the needs of clinical evaluation of the prognosis of NSCLC.

Increasing research identified thousands of regulatory non-coding RNAs, which can be further classified with short noncoding transcripts (fewer than 200 nucleotides) and long noncoding RNAs (lncRNAs; greater than 200 nucleotides) based on transcript length [5]. With the development of research on the treatment of tumors, it is demonstrated that lncRNAs act as crucial roles in cell biology via regulating various processes, such as post-transcriptional processing and transcription, cellular activities [6, 7]. In the field of cancer, compared with the normal tissues, lncRNAs expression was dysregulated in tumor cells and tissues and was widely involved in the malignant transformation of tumor cells, epigenetic modification, and other biological processes [8, 9]. Numerous abnormally expressed lncRNAs were correlated with the survival of LUSC patients and involved in the pathogenesis of LUSC, such as linc p53-induced transcript (linc-PINT) [10] and PSMG3-antisense 1 (PSMG3-AS1) [11].

The aberrant expression of linc02544 was observed in several diseases, such as hypertrophic scars [12] and breast cancer [13]. A recent study identified numerous lncRNAs and mRNAs in lung adenocarcinoma, among which include linc02544 [14]. Whereas, whether linc02544 has abnormal expression in LUSC and plays a regulatory role in LUSC remains unclear. The expression of linc02544 was detected in LUSC tissues and cell lines, as well as assessed the relation between the linc02544 levels and outcomes of the patients. Subsequently, we experimentally explored the influence of linc02544 on the cellular activities of LUSC cells.

## Materials and methods

### Clinical patients samples

There were 131 patients with a definite pathological diagnosis of LUSC who received surgical treatment in this study from 2014 to 2016. The participants did not receive any anti-tumor therapy prior to the operation. Paired adjacent normal tissues and tumor tissues were obtained from the 131 patients during operation and were frozen in liquid nitrogen after collection. The clinical parameters of patients were collected and recorded from medical records. Besides, the patients received a five-year survival follow-up survey.

This study was reviewed and approved by the Ethics Committee of the Jinling Hospital, Nanjing University School of Medicine, and was conducted following the Declaration of Helsinki. All participants signed written informed consent prior to study inclusion.

### Cell lines and cell culture

Human LUSC cells SK-MES-1 (HTB-58), NCI-H226 (H226; CRL-5826), NCI-H1703 (H1703; CRL-5889), and NCI-H520 (H520; HTB-182) and normal lung epithelial cell (BEAS-2B; CRL-9609) were acquired from the ATCC (Manassas, VA, USA). All LUSC cells were cultured in DMEM (Gibco, CA, USA) with 10% FBS (Gibco) at 37 °C in a humidified incubator.

### Cell transfection

The siRNA oligonucleotide for linc02544 (si-linc02544), siRNA negative control (si-RNA), miR-138-5p mimic, and mimic NC were designed by GenePharma (Shanghai, China). Cell transfection was conducted in LUSC cells at roughly 70% confluence with the help of Lipofectamine 2000 reagents (Invitrogen, CA, USA). Then the cells were cultured for 24 h and RT-qPCR was used to detect the efficiency.

### RNA extraction and RT-qPCR

Total RNA was isolated from tissues and cells using RNeasy Plus mini kit (Qiagen). M-MLV Reverse Transcriptase (Promega, USA) was used for reverse transcription to form the first-strand cDNA. qPCR was performed using SYBR Green PCR Master Mix (Applied Biosystems). The relative expression of linc02544 or miR-138-5p was calculated by the ΔΔCt method (fold expression = 2^−ΔΔCt^) that was normalized to the GAPDH or U6 expression.

### Cell counting kit-8 assay

The cell counting kit-8 (CCK-8; Dojindo, Japan) assay was carried out to measure the cell proliferation abilities [15]. The transfected SK-MES-1 and NCI-H520 cells (3000 cells/well) were cultured for 0, 24, 48, 72 h in 96-well plates. The 10 µl CCK-8 solution was added to the medium and incubated for 2 h at 37 °C. Then the absorbance (450 nm) was measured by a microplate reader.

### Transwell assay

Cell migration or invasion capacities were determined by the Transwell migration or invasion assays, respectively. The cells in an FBS-free culture medium were inoculated onto an uncoated matrigel and a Matrigel (concentration of 1 mg/ml; duration of coating 3 h) coating polycarbonate membrane filter in a transwell chamber (8 μm pore size; Corning). The bottom chamber was added with the culture medium with 10% FBS. After 24 h, the migrated or invaded cells through the membrane were stained and were recorded in five random field images under a light microscope (Olympus).

### Western blot assay

Cells were washed with PBS buffer and lysed in RIPA lysis buffer (Beyotime) on ice to extract total protein. The BCA Protein Assay Kit (Pierce) was used to quantify the proteins. Then cell extracts were separated on a 10% SDS-polyacrylamide gel and then transferred onto PVDF membranes (Millipore, USA). Primary antibodies (anti-MMP-2, anti-MMP-9, anti-e-cadherin, anti-vimentin, and anti-GAPDH; Abcam) were prepared and incubated with the membrane overnight. Membranes were washed and incubated with HRP-conjugated secondary antibody (goat anti-rabbit IgG) for 1 h. Finally, protein signals were visualized and quantified using enhanced chemiluminescence (ECL) detection and Image Lab™ Software (Bio-Rad, China).

### Dual-luciferase reporter assay

*In silico* prediction analysis for linc02544 and E2F3 binding sites with miR-138-5p were predicted with LncBase Experimental v.2 (http://carolina.imis.athena-innovation.gr/diana_tools/web/index.php?r=lncbasev2%2findex-experimental) and TargetScan (www. targetscan.org) online databases. To construct luciferase reporter vectors, the 3’-UTR fragment of linc02544 containing the predicted potential binding sites of miR-138-5p was cloned into pmiR-RB-REPORT luciferase reporter vector (RiboBio, Guangzhou, China), and named linc02544 WT. The linc02544 mutant sequence was also cloned into the vector and named linc02544 MUT. The E2F3 WT and MUT luciferase reporter vectors were constructed. To confirm the relationship among linc02544, miR-138-5p, and E2F3, the linc02544 WT or linc02544 MUT construct, and E2F3 WT or E2F3 MUT was cotransfected with miR-138-5p mimic or mimic NC into LUSC cells using Lipofectamine 2000 (Invitrogen) for 24 h, respectively. The Firefly luciferase activities were detected using the Dual-luciferase Reporter Assay System (Promega, WI, USA) and were normalized against Renilla luciferase activities.

### Statistical analysis

The statistical data analysis was handled with SPSS version 20.0 (SPSS software, Chicago, IL, USA) and GraphPad version 7.0 (GraphPad Software, San Diego, CA, USA). Data represent mean values and standard deviations (SD) obtained from at least three independent experiments. Data comparisons were performed using a Student’s t-test, a one-way analysis of variance (ANOVA), or two-way ANOVA. Pearson’s linear regression analysis was used to analyze the correlation between linc02544 and miR-138-5p expression. The clinical prognostic significance of linc02544 was validated using the Kaplan-Meier curve analysis and compared using the log-rank test. Statistical significance was defined when *P < 0.05, **P < 0.01, ***P < 0.001.

## Results

### Linc02544 is overexpressed in LUSC tissues and in different clinical parameters of patients

**Fig. 1:**
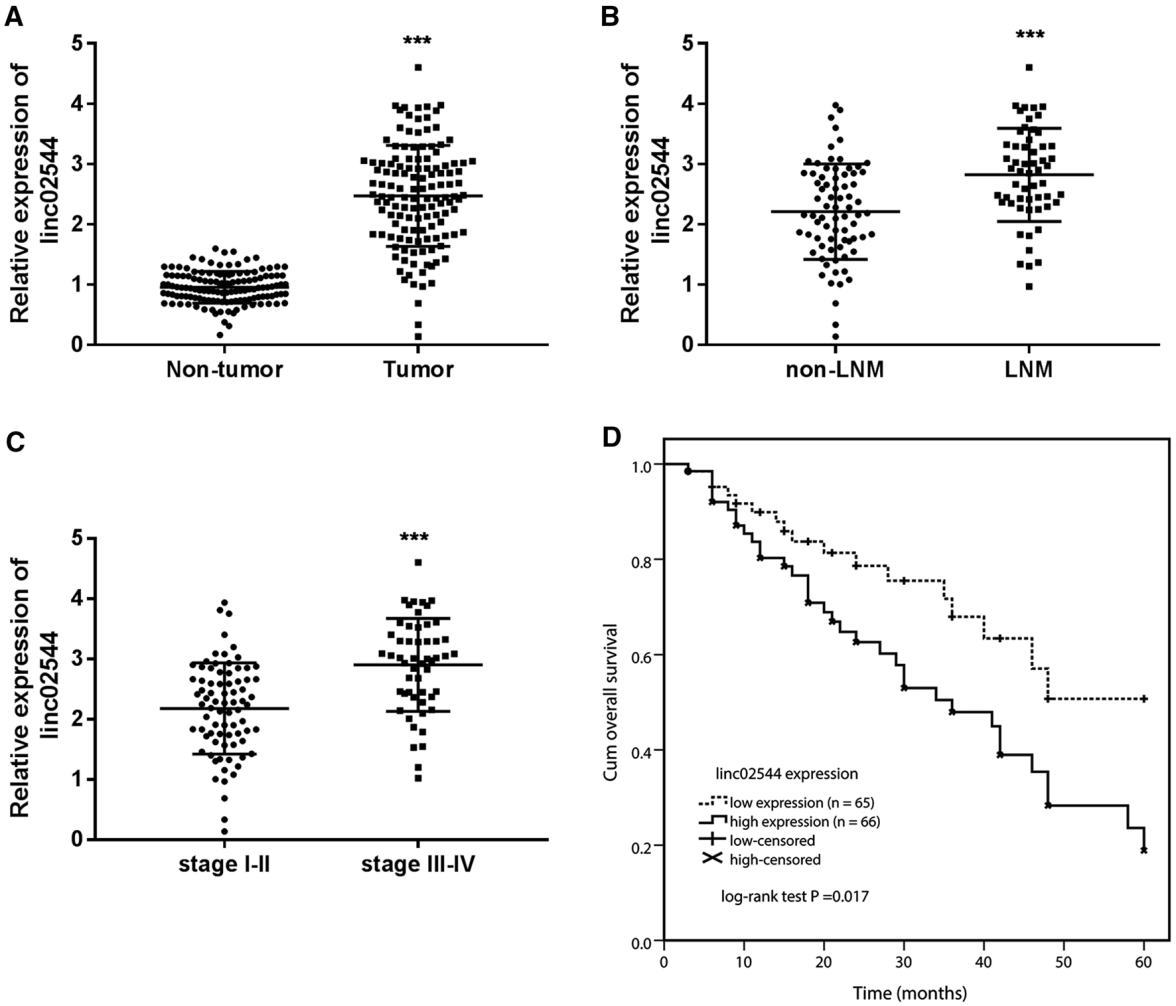
Linc02544 was increased in LUSC tissue samples and predicted poor outcomes. **A** The linc02544 levels in LUSC tissue samples were higher than in adjacent non-tumor tissue samples. **B** linc02544 relative expression in tumor tissues of patients without LNM and patients with LNM. **C** linc02544 expression levels were higher in the tissues of patients at TNM stage III-IV than that in patients at stage I-II. ***P < 0.001. **D** Kaplan-Meier curve of linc02544 in LUSC. Log-rank test *P* = 0.017

Firstly, the expression of linc02544 in LUSC tissue samples was identified by RT-qPCR. The data in Fig. 1 A revealed that linc02544 expression levels were increased in tumor tissues (*P* < 0.001). Then, for analyzing the relationship between linc02544 and patients’ clinical characteristics, the patients were divided into the low linc02544 expression group (n = 65) and high linc02544 expression group (n = 66) based on the median linc02544 expression level in tumor tissues. Table 1 revealed that high linc02544 expression was correlated with TNM stage (*P* = 0.003) and lymph node metastasis (*P* = 0.017). The expression of linc02544 was further analyzed in the tissues of patients with lymph node metastasis (LNM) and without lymph node metastasis (non-LNM), and the results indicated that linc02544 expression levels were higher in LNM tissues than non-LNM tissues (*P* < 0.001; Fig. 1B). Moreover, the expression levels of linc02544 were higher in the tissues of patients at high TNM stage (III-IV) than in the tissues of patients at the early stage (I-II) (*P* < 0.001, Fig. 1C). The above results confirmed the relationship between linc02544 expression and lymph node metastasis and the TNM stage. The tumor size (*P* = 0.066) and grade (*P* = 0.053) have no significant relationship with linc02544, which may be affected by the sample size. Whereas, the age, sex, and smoking history of the patients have no statistical significance (*P* > 0.05, Table 1).

**Table 1:** Correlation of the linc02544 expression with clinical parameters in patients with LUSC

Parameters	Cases (n = 131; %)	Linc02544 expression		*P-value*
		Low (n = 65; %)	High (n = 66; %)	
Age				0.136
≤ 60	68 (51.91)	38 (58.46)	30 (45.45)	
> 60	63 (48.09)	27 (41.54)	36 (54.55)	
Sex				0.545
Female	57 (43.51)	30 (46.15)	27 (40.91)	
Male	74 (56.49)	35 (53.85)	39 (59.09)	
Tumor size				0.066
≤ 5 cm	66 (50.38)	38 (58.46)	28 (42.42)	
> 5 cm	65 (49.62)	27 (41.54)	38 (57.58)	
TNM stage				
I–II	78 (59.54)	47 (72.31)	31 (46.97)	0.003
III–IV	53 (40.46)	18 (27.69)	35 (53.03)	
Lymph node metastasis				
Negative	75 (54.25)	44 (67.69)	31 (46.97)	0.017
Positive	56 (42.75)	21(32.31)	35 (53.03)	
Smoking history				
No	61(46.56)	26 (40.00)	35 (53.03)	0.135
Yes	70(53.44)	39 (60.00)	31 (46.97)	
Grade				0.053
Well + Moderate	84 (64.12)	47 (72.31)	37 (56.06)	
Poor	47 (35.88)	18 (27.69)	29 (43.94)	

### High linc02544 expression predicted patients’ unfavorable survival rate

The clinical prognostic significance of linc02544 was also evaluated using the Kaplan-Meier curve and compared using a log-rank test. The survival curve of Fig. 1D displayed that patients with high linc02544 expression exhibited worse overall survival rate compared with the low linc02544 expression group (log-rank test *P* = 0.017). The multivariate Cox proportional hazard regression model indicated that only high linc02544 expression (HR = 2.208, 95%CI: 1.181–4.127, *P* = 0.013), positive lymph node metastasis (HR = 2.014, 95%CI: 1.107–3.665, *P* = 0.022), and high TNM stage (HR = 2.164, 95%CI: 1.081–4.330, *P* = 0.029) were significant markers of the deterioration of overall survival (Table 2).

**Table 2:** Multivariate Cox analysis of clinical characteristics in relation to overall survival

Parameters	Multivariate analysis		
	HR	95% CI	*P*
Linc02544	2.208	1.181–4.127	0.013
Age	1.576	0.883–2.811	0.124
Sex	1.333	0.725–2.453	0.355
Tumor size	1.495	0.822–2.720	0.187
TNM stage	2.014	1.107–3.665	0.022
Lymph node metastasis	2.164	1.081–4.330	0.029
Smoking history	1.229	0.671–2.253	0.504
Grade	1.767	0.924–3.380	0.085

### si-linc02544 restrained cellular behaviors in vitro

**Fig. 2:**
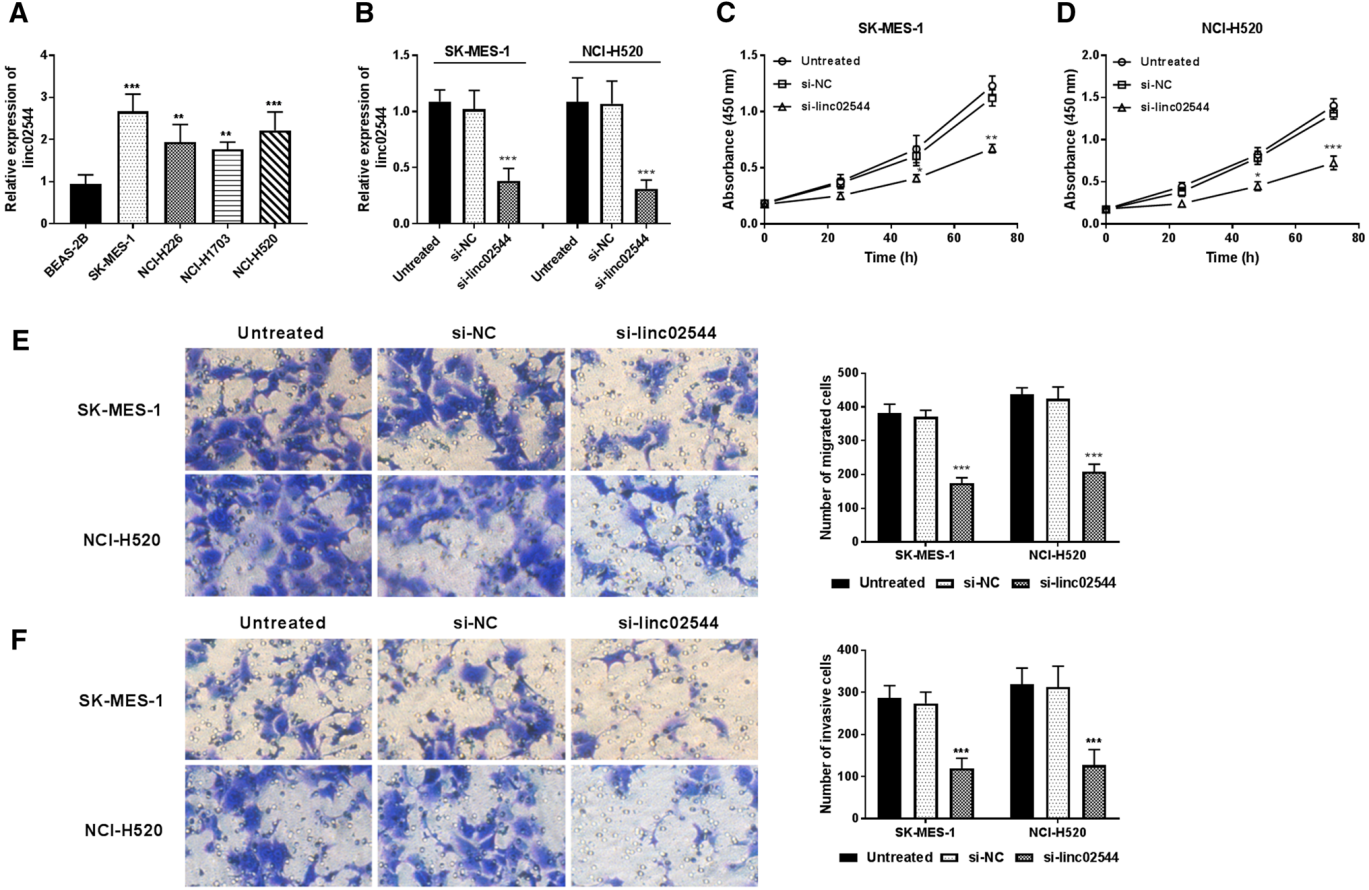
linc02544 regulated LUSC cellular activities. **A** The linc02544 levels were higher in LUSC cells than in normal epithelial lung BEAS-2B cells. **B** The expression of linc02544 was downregulated by si-linc02544 after transfection of si-linc02544. The cell proliferation was inhibited by linc02544 downregulation in SK-MES-1 cells (**C**) and NCI-H520 cells (**D**). **E** The cell migratory abilities were suppressed by si-linc02544 in both SK-MES-1 and NCI-H520 cells (200 ×, magnification). **F** Downregulation of linc02544 inhibited LUSC cell invasive capacities in both SK-MES-1 and NCI-H520 cells (200 ×, magnification). *P < 0.05, **P < 0.01, ***P < 0.001

First, we measured basal levels of linc02544 in a normal lung epithelial cell line BEAS-2B and four human LUSC cell lines SK-MES-1, NCI-H226, NCI-H1703, and NCI-H520 by RT-qPCR. As presented in Fig. 2A, the levels of linc02544 were higher in the LUSC cell lines than in normal BEAS-1B cells (*P* < 0.01). To explore whether linc02544 expression has a crucial role in cell growth and migration by knockdown of linc02544 using si-linc02544. The linc02544 was downregulated by si-linc02544 in SK-MES-1 and NCI-H520 cells (*P* < 0.001, Fig. 2B). The cell proliferation was weakened by si-linc02544 in both SK-MES-1 (*P* < 0.05, Fig. 2C) and NCI-H520 cells (*P* < 0.05, Fig. 2D). The transwell migration (Fig. 2E) and invasion (Fig. 2F) assay results showed that there was a significant reduction in cell migration and invasion abilities in SK-MES-1 and NCI-H520 cells (*P* < 0.001).

**Fig. 3:**
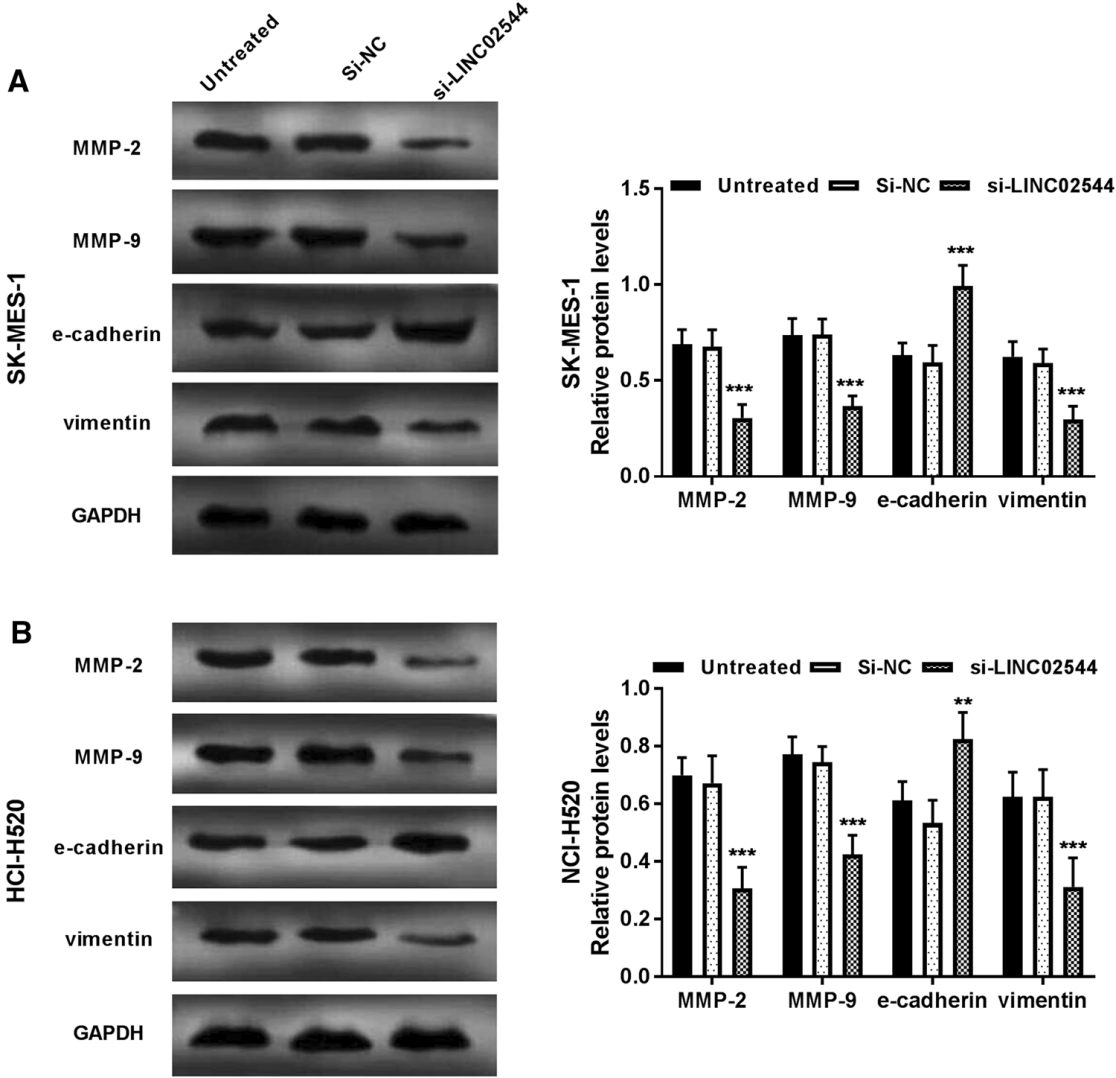
The metastasis-related protein levels were measured using a Western blot assay. **A** and **B** The MMP-2, MMP-9, and vimentin levels were decreased while E-cadherin levels were increased in cells transfected with si-linc02544 compared to untreated cells. **P < 0.01, ***P < 0.001

Moreover, metastasis-related proteins MMP-2, MMP-9, E-cadherin, and vimentin were measured by Western blot analysis. Expression of MMP-2, MMP-9, and vimentin was decreased while E-cadherin was increased in si-linc02544 cells compared with that in untreated cells (*P* < 0.01, Fig. 3A and 3B). The original WB picutures were listed in Additional files 1 and 2. These data indicated silence of linc02544 was sufficient to recede migration and invasion by decreasing the expression of MMP-2, MMP-9, and vimentin while increasing E-cadherin in LUSC cells.

### miR-138-5p was a target of linc02544

**Fig. 4:**
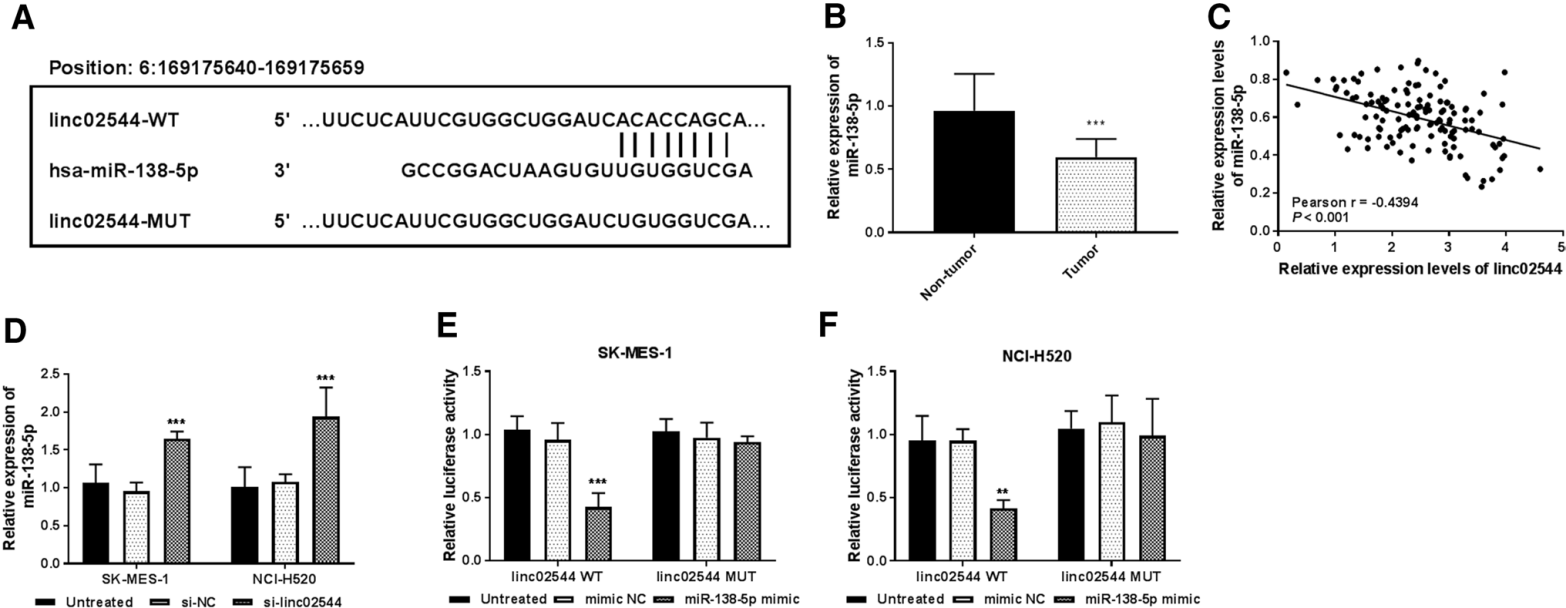
miR-138-5p, a target of linc02544. **A** The putative binding sites between linc02544 and miR-138-5p. **B** The miR-138-5p levels in LUSC tissues and corresponding adjacent non-tumor tissues were measured by RT-qPCR. **C** Pearson’s linear regression analysis confirmed the negative correlation between linc02544 expression and miR-138-5p (r = − 0.4394, *P* < 0.001). **D** The expression of miR-138-5p was increased in SK-MES-1 and NCI-H520 cells after transfecting with si-linc02544. The interaction between linc02544 and miR-138-5p was determined by luciferase reporter assay in SK-MES-1 (**E**) and NCI-H520 cells (**F**). **P < 0.01, ***P < 0.001

The online publically database lncBase v.2 identified putative miRNAs in the linc02544 3’-UTR. miR-138-5p has identified the binding sites with linc02544 3’-UTR at the position of 6:169175640 to 169,175,659 (Fig. 4A). Then miR-138-5p levels were detected in tumor tissues and the results showed that miR-138-5p levels were lower in tumor tissues than in non-tumor tissues (*P* < 0.001, Fig. 4B). Then there is a negative correlation between linc02544 and miR-138-5p expression levels in tumor tissues (Pearson r = -0.4394, *P* < 0.001, Fig. 4C). In contrast, the miR-138-5p levels were higher in cells transfected with si-LINC02544 than that in untreated SK-MES-1 and NCI-H520 cells (*P* < 0.001, Fig. 4D). To confirm the relationship between linc02544 and miR-138-5p, the dual-luciferase reporter assay indicated that the luciferase activities were repressed by a miR-138-5p mimic in cells cotransfected with miR-138-5p mimic and linc02544 WT, while no changes were observed in cells cotransfected with miR-138-5p mimic and linc02544 MUT in both SK-MES-1 (p < 0001, Fig. 4E) and NCI-H520 cells (*P* < 0.01, Fig. 4F). The data displayed that miR-138-5p was a target of linc02544.

### E2F3 may be the potential target of miR-138-5p

**Fig. 5:**
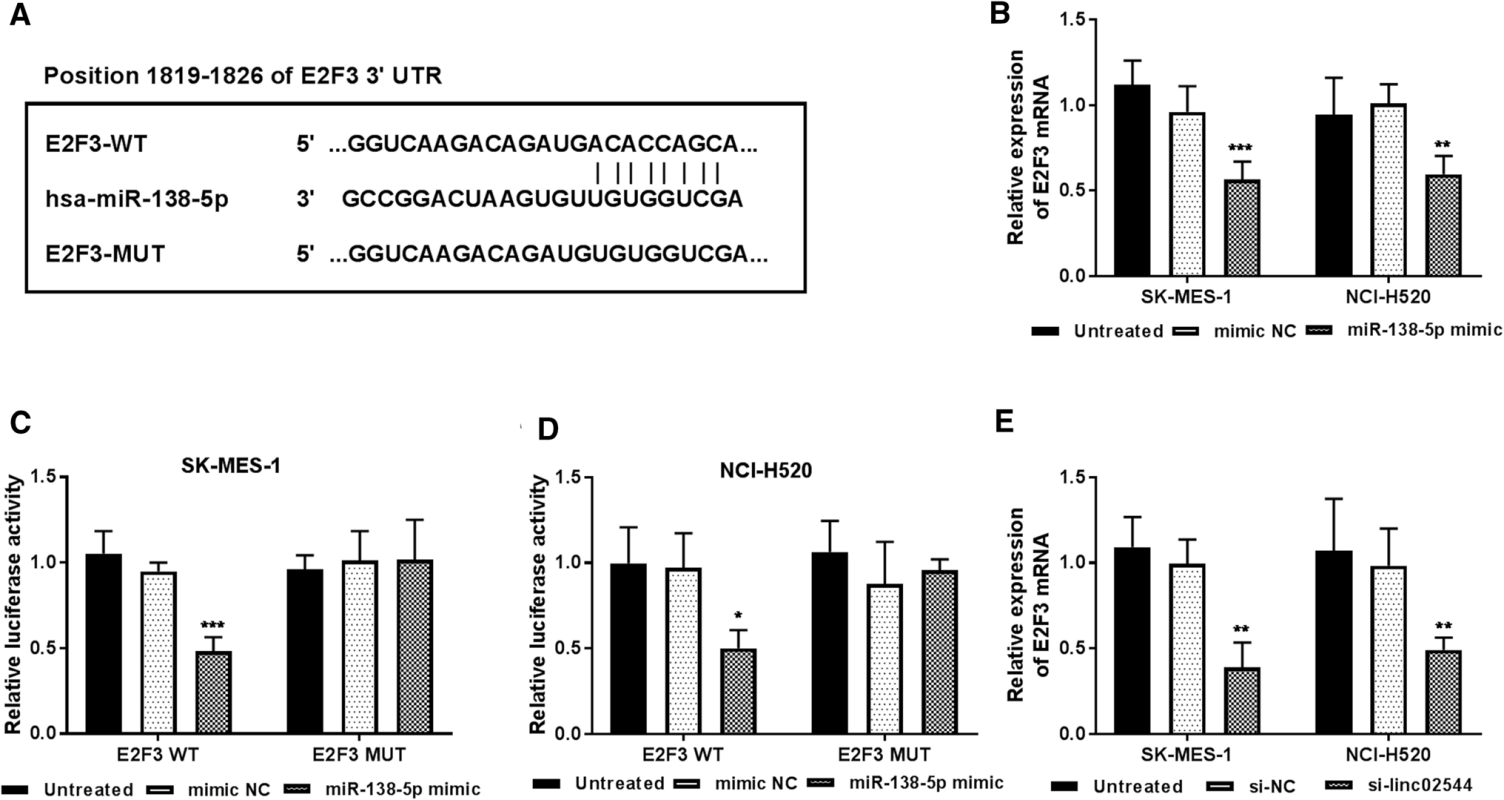
E2F3 may be a downstream target of miR-138-5p. **A** The binding sites between miR-138-5p and E2F3. **B** Overexpression of miR-138-5p decreased the E2F3 mRNA levels compared with that in untreated cells. **C** and **D** miR-138-5p repressed the luciferase activities of E2F3 WT-3’-UTR but no effect on the E2F3 MUT-3’-UTR. **E** The E2F3 mRNA levels were decreased in cells transfected with si-linc02544 compared that in untreated cells. *P < 0.05, **P < 0.01, ***P < 0.001

LncRNA-miRNA-mRNA interactions are generally related to various biological processes of diseases. Thus, we predicted potential targets of miR-138-5p using the TargetScan database (www.targetscan.org). Among these targets, E2F3 has a predicted targeting binding site of miR-138-5p (Fig. 5A), which was upregulated in lung cancer [16, 17]. We speculate that E2F3 may be a downstream target of miR-138-5p. To test this hypothesis, the effect of miR-138-5p on E2F3 mRNA was determined. We found that overexpression of miR-138-5p decreased E2F3 mRNA expression (*P* < 0.01, Fig. 5B). Furthermore, miR-138-5p repressed the luciferase activities of E2F3 WT-3’-UTR but no effect on the E2F3 MUT-3’-UTR (*P* < 0.05, Fig. 5C and 5D). Moreover, we observed the expression of E2F3 mRNA was decreased following the downregulation of linc02544 (*P* < 0.01, Fig. 5E). Together, these data suggest that linc02544 may act as a ceRNA of miR-138-5p by regulating E2F3.

## Discussion

LUSC, as one of the main pathological subtypes of NSCLC, is considered to be among the commonest malignant tumors with high mortality and metastasis rate [18]. lncRNAs have been demonstrated to act essential roles in regulating tumor progression and initiation, tumor metastasis, which are often deemed putative biomarkers and therapeutic targets for the treatment of tumors [19, 20].

The abnormal expression of linc02544 was reported in several diseases [12, 13]. For instance, a study performed RNA sequencing and acquired many differently expressed lncRNAs, mRNAs, and circRNAs in the human hypertrophic scar, in which linc02544 was one of the top three deregulated expression lncRNAs, which may play a central role in hypertrophic scar [12]. Linc02544, a full-length 544 nt non-coding RNA, is highly expressed in breast cancer and may be an application prognostic marker for neoadjuvant therapy patients with breast cancer [21]. The abnormal expression and functionality of linc02544 have only been reported in lung adenocarcinoma [14]. The current study also observed the upregulation of linc02544 in LUSC, which suggests that linc02544 may have a promoting role in LUSC.

LncRNAs are reported to have obvious tissue-specific expression and are associated with patients’ outcomes, which may have diagnostic or prognostic values [22, 23]. For instance, lncRNA HOTTIP was upregulated in lung squamous cell carcinoma and was considered an independent prognostic marker in early-stage non-small cell lung cancer [22]. In addition, the high expression of linc02544 was also observed in patients with lymph node metastasis and high stage, which implied that linc02544 expression may be associated with the survival of the patients with LUSC. The clinical analysis results also indicated that high linc02544 expression has a correlation with lymph node metastasis and TNM stage, which suggests we explore its clinical significance with prognosis. The results revealed that high linc02544 expression may be an indicator of shorter overall survival, suggesting the potential significance of linc02544 as a prognostic biomarker for patients with LUSC.

Relevant studies have indicated that lncRNAs can exert cancer-suppressing or cancer-promoting effects on cellular activities in a variety of cancer types [24, 25]. For instance, lncRNA HOX transcript antisense intergenic RNA (HOTAIR) was highly expressed in breast ductal carcinoma and behaves as a promoting regulator of tumor cell migration and growth in early-stage breast cancer [26]. In this study, linc02544 was also increased in LUSC cells. Interestingly, the relationship between the phenotype of cancer cell lines does not correlate with the data derived from patients’ tissue samples, where the advanced stages of disease progression such as lymph node metatasia and TNM stage showe higher expression of linc02544 compared to less aggressive sample. The observation was interesting. The expression of linc02544 in tumor tissues was observed in 136 paired tissues with three repeats, while its expression in tumor cell lines was measured five times, which may be biased. On the other hand, there may be some differences in expression in tissues and in vitro cell lines, which exists in discrepancy. Bring forward, weakening linc02544 expression could bring down the cell proliferation, invasion, and migration of LUSC cells. Moreover, metastasis-related protein levels of MMP-2, MMP-9, and vimentin were decreased while E-cadherin was increased in si-linc02544 cells compared with that in untreated cells. These data suggest that silence of linc02544 was sufficient to recede migration and invasion by decreasing the expression of MMP-2, MMP-9, and vimentin while increasing E-cadherin in LUSC cells. These results hinted that linc02544 knockdown could be considered a putative biomarker for the treatment of LUSC.

LncRNAs could regulate tumor cell activities through interacting 3’-UTR of miRNAs in the competitive endogenous RNA regulatory networks (lncRNAs-miRNAs-mRNAs) [27, 28]. For example, lncRNA LOC100507144 could contribute to colorectal cancer progression and metastasis through regulating CD44/Nanog/Sox2/miR-302/miR-21 axis [24]. To underlying the potential mechanism of linc02544 in LUSC, bioinformatics analysis predicted miR-138-5p as a candidate miRNA. miR-138-5p has been reported to be a tumor suppressor in prostate cancer [29], gastric cancer[30], and pancreatic cancer [31]. In lung cancer, miR-138-5p inhibition could promote carcinogenesis and progression by targeting E2F3, CDK8, ZEB2, PD-L1, and PD-1 [32–35], and the present results displayed that miR-138-5p is downregulated in LUSC tissues and cells. Correlation analysis revealed that miR-138-5p directly interacts with linc02544, and linc02544 negatively regulates miR-138-5p. Moreover, the interaction between miR-138-5p and E2F3 was also validated. E2F3 mRNA was highly expressed in lung cancer tissues and associated with poor survival outcomes for the patient, as well as promoted cell proliferation, migration, and invasion [16, 17, 36]. Thus, these data revealed that linc02544 may be involved in regulating LUSC cell proliferative abilities, invasive capacities, and migratory potential through negatively regulating miR-138-5p and regulating E2F3 expression.

In summary, linc02544 was upregulated in LUSC samples by analyzing 131 datasets. The linc02544 expression was higher in positive lymph node metastasis and high TNM stage LUSC samples. The high expression of linc02544 was related to a shorter overall survival time in patients with LUSC and may be a putative prognostic marker for patients with LUSC. Moreover, the silence of linc02544 may restrain LUSC cellular activities by regulating miR-138-5p/E2F3. These results may provide a prospective clinical trial for the prognosis and treatment of patients with LUSC.

## Supplementary Information

Below is the link to the electronic supplementary material.

Supplementary file 1 (ZIP 2719 KB)

Supplementary file 2 (ZIP 6838 KB).

## Data Availability

Data for this study can be obtained by contacting the corresponding author.
