# Supplementary figures and images for: Upregulation of long noncoding RNA linc02544 and its association with overall survival rate and the influence on cell proliferation and migration in lung squamous cell carcinoma
Source: Discov Oncol. 2022 May 30;13:41. doi: 10.1007/s12672-022-00501-5 (PMC9151984; doi:10.1007/s12672-022-00501-5)

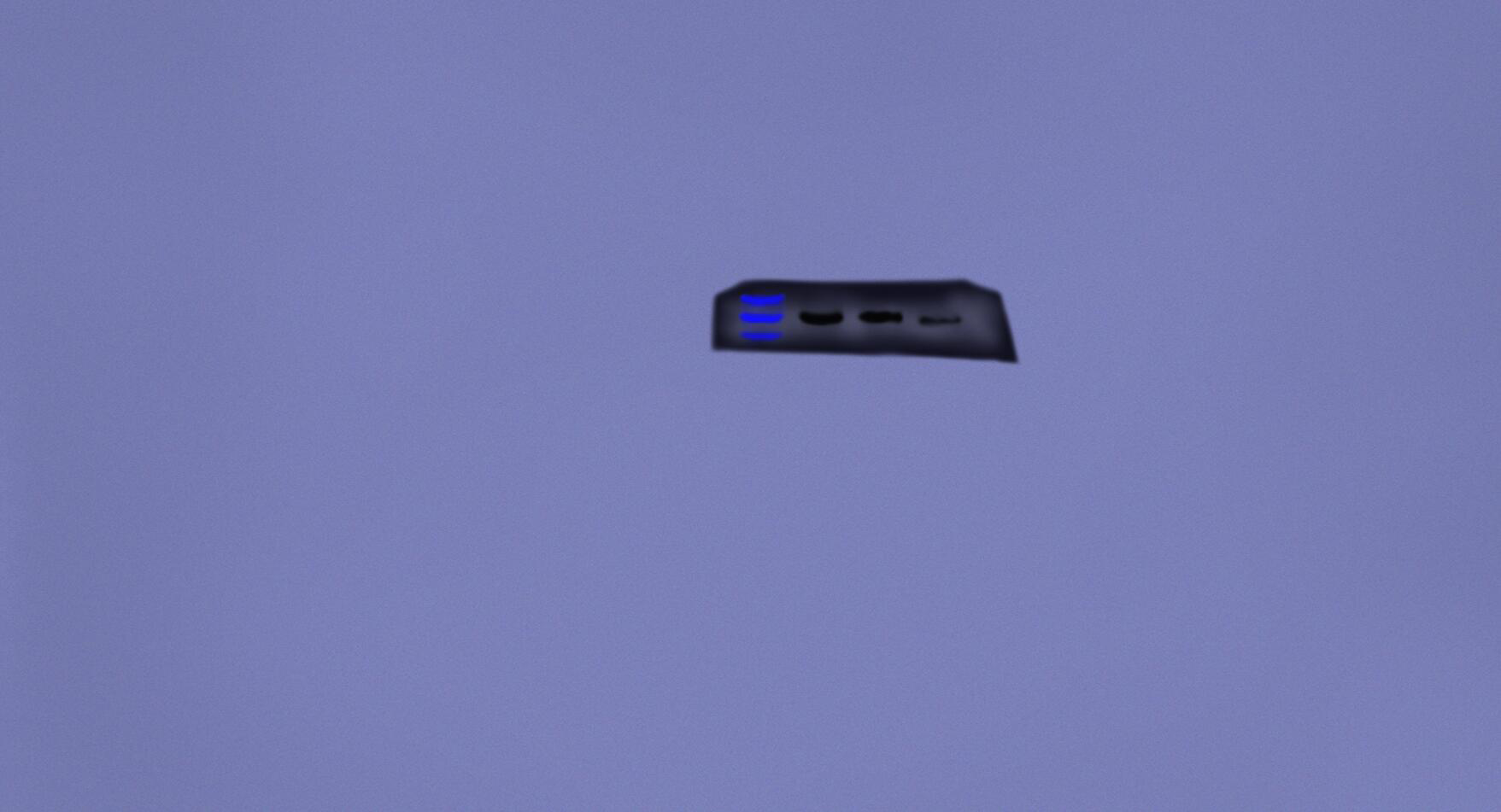

Supplement: Supplementary file 1 — (ZIP 2719 KB) [file 12672_2022_501_MOESM1_ESM.zip › HCI-H520-WB/1-MMP-2.tif]

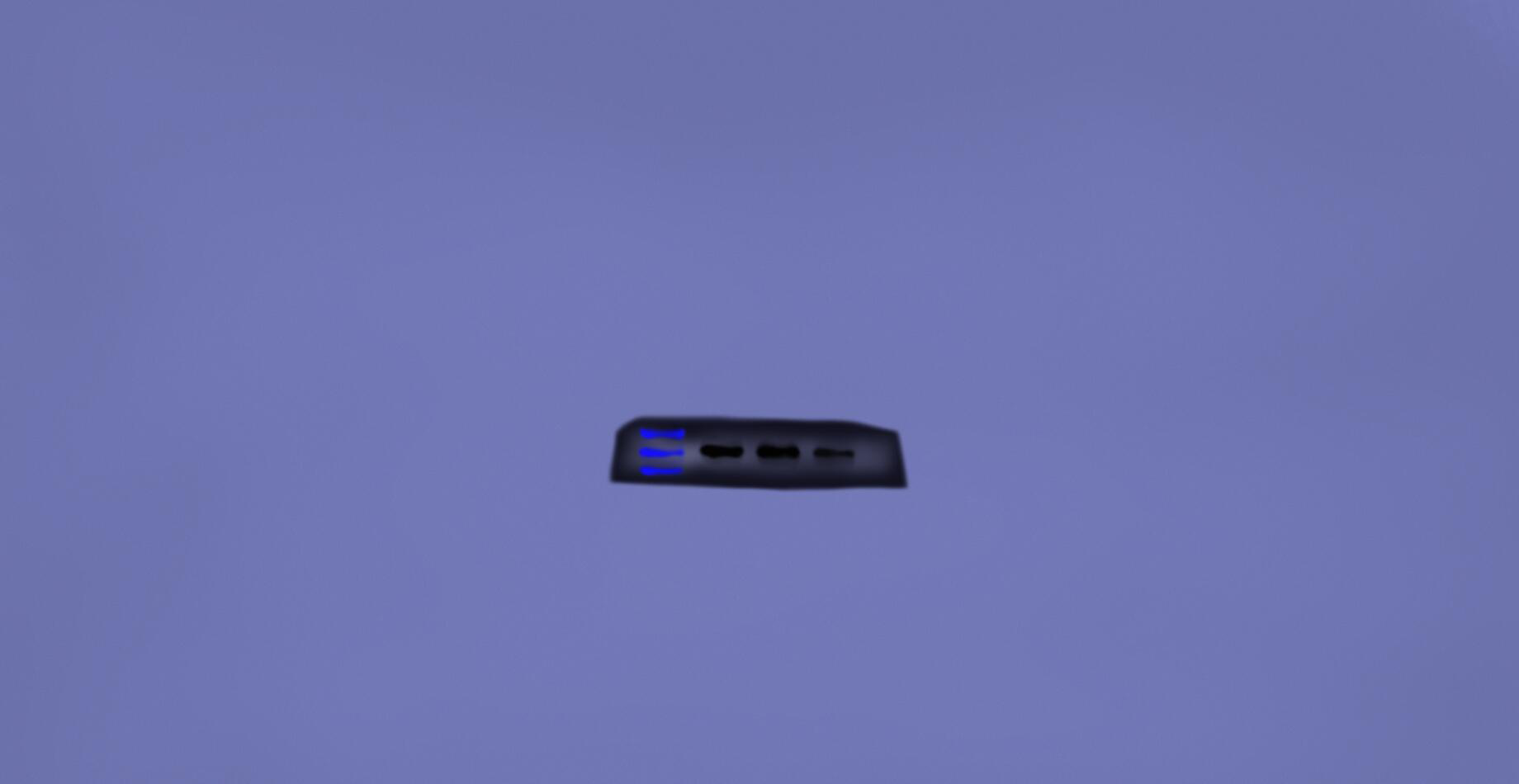

Supplement: Supplementary file 1 — (ZIP 2719 KB) [file 12672_2022_501_MOESM1_ESM.zip › HCI-H520-WB/2-MMP-9.tif]

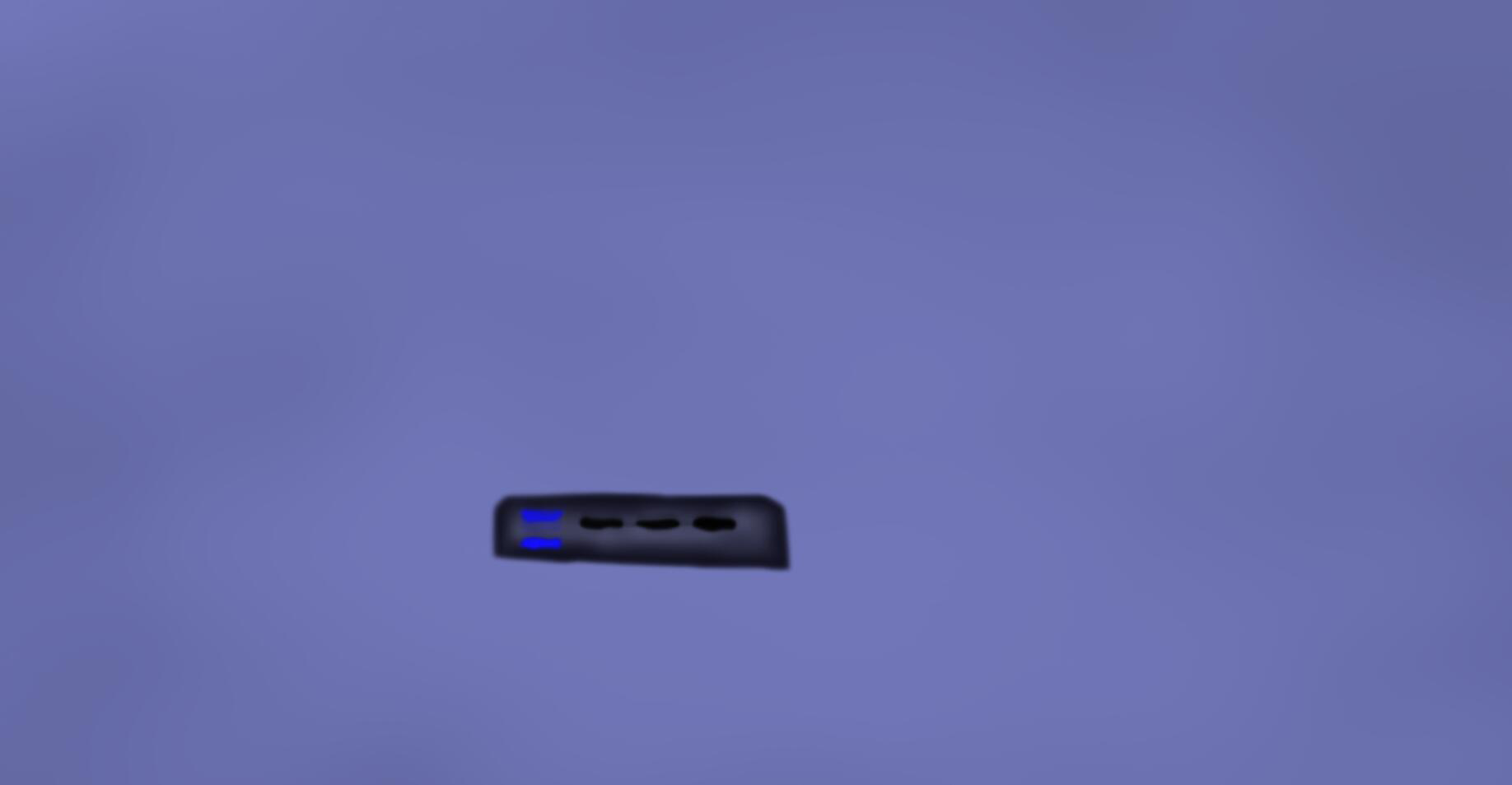

Supplement: Supplementary file 1 — (ZIP 2719 KB) [file 12672_2022_501_MOESM1_ESM.zip › HCI-H520-WB/3-E-cadherin.tif]

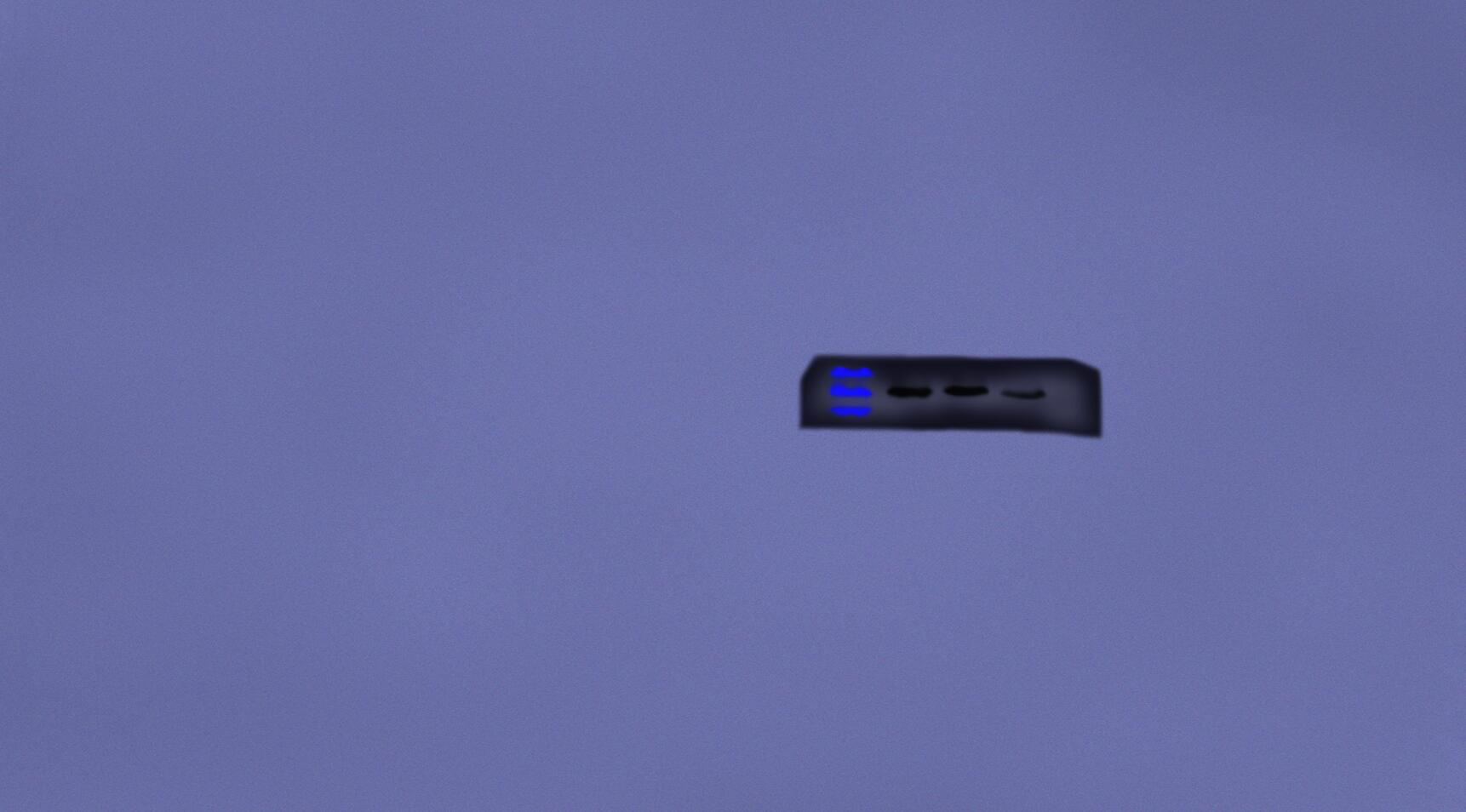

Supplement: Supplementary file 1 — (ZIP 2719 KB) [file 12672_2022_501_MOESM1_ESM.zip › HCI-H520-WB/4-Vimentin.tif]

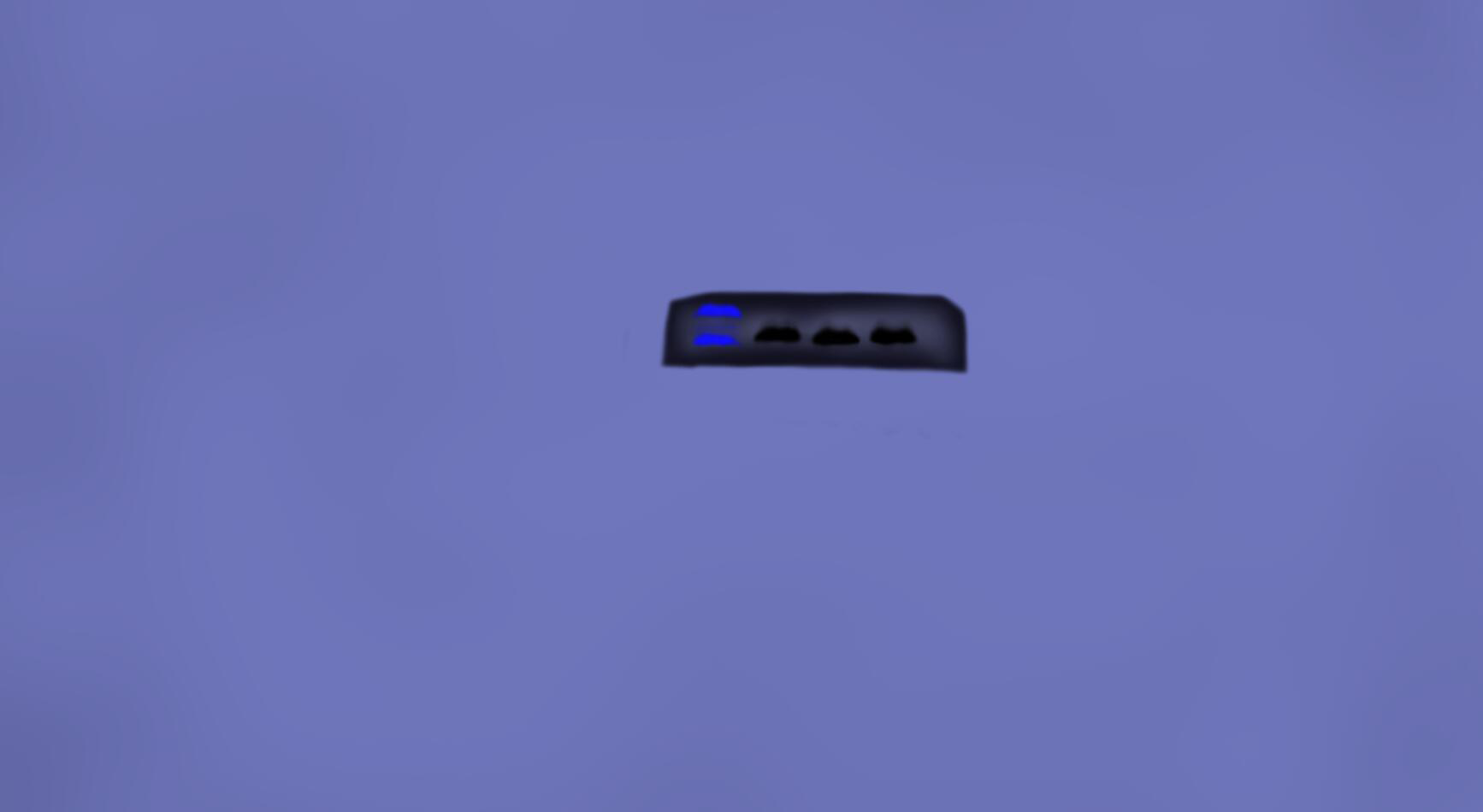

Supplement: Supplementary file 1 — (ZIP 2719 KB) [file 12672_2022_501_MOESM1_ESM.zip › HCI-H520-WB/5-GAPDH.tif]

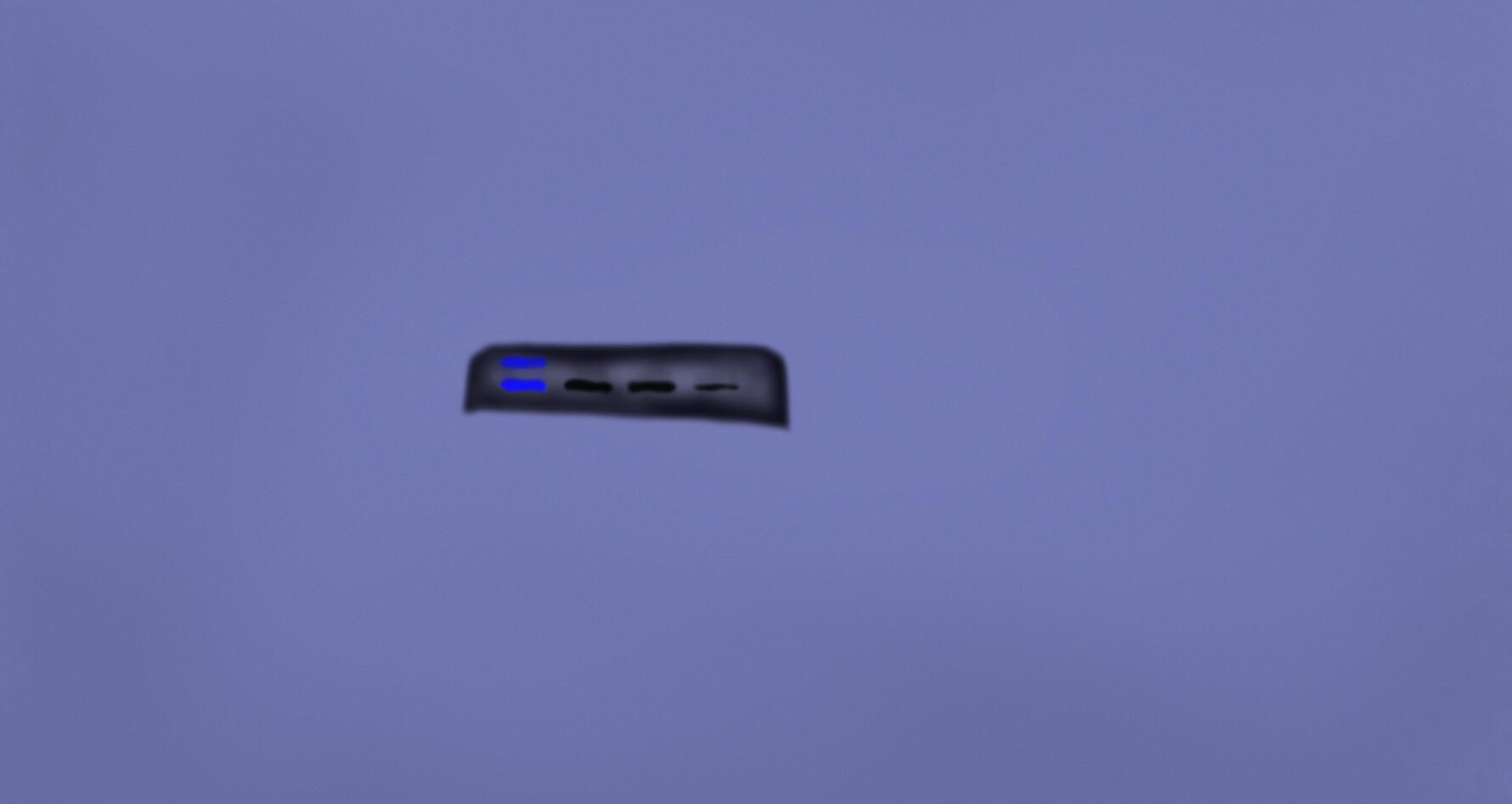

Supplement: Supplementary file 2 — (ZIP 6838 KB). [file 12672_2022_501_MOESM2_ESM.zip › SK-MES-1-WB/1-MMP-2.tif]

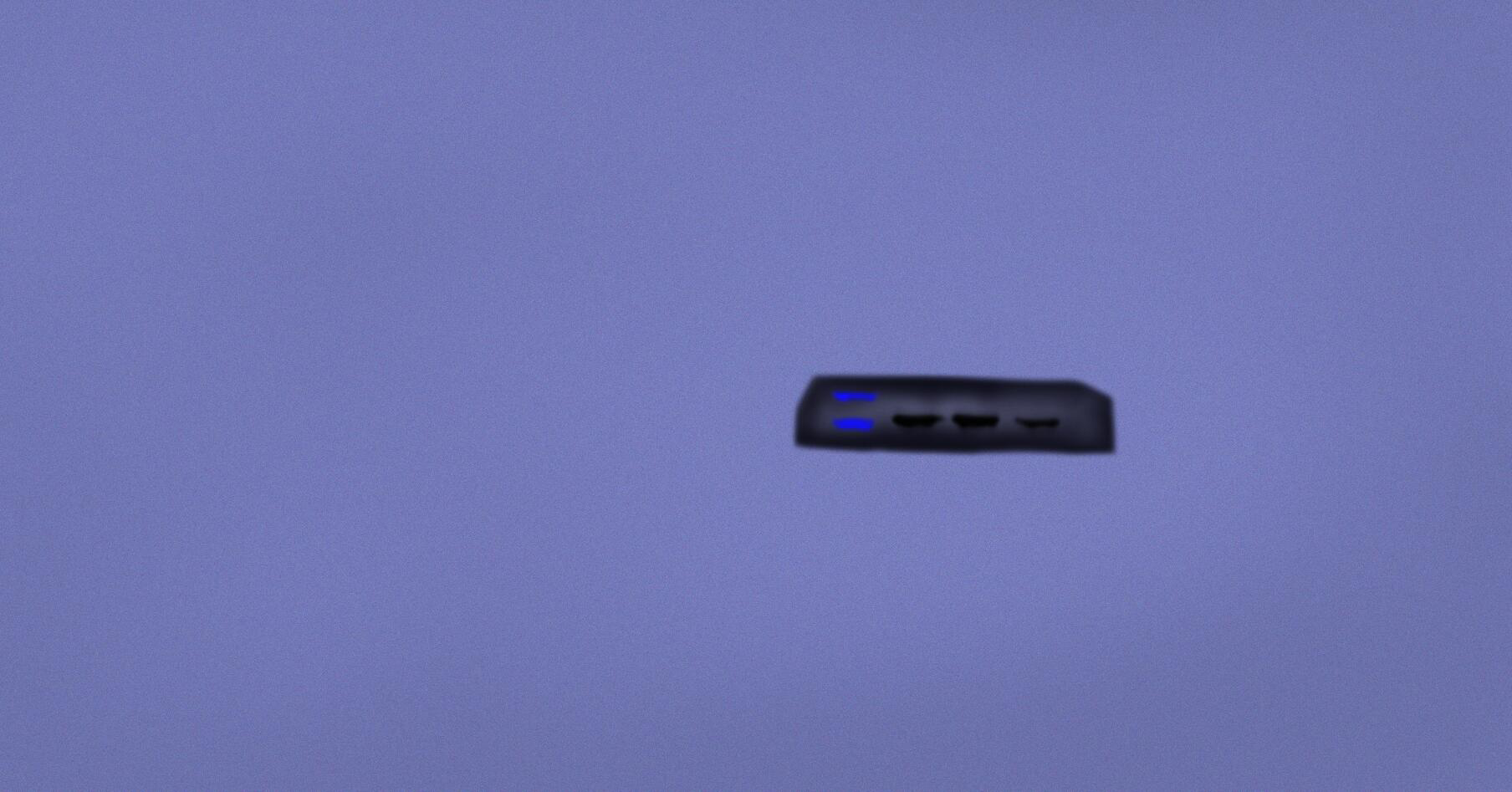

Supplement: Supplementary file 2 — (ZIP 6838 KB). [file 12672_2022_501_MOESM2_ESM.zip › SK-MES-1-WB/2-MMP-9.tif]

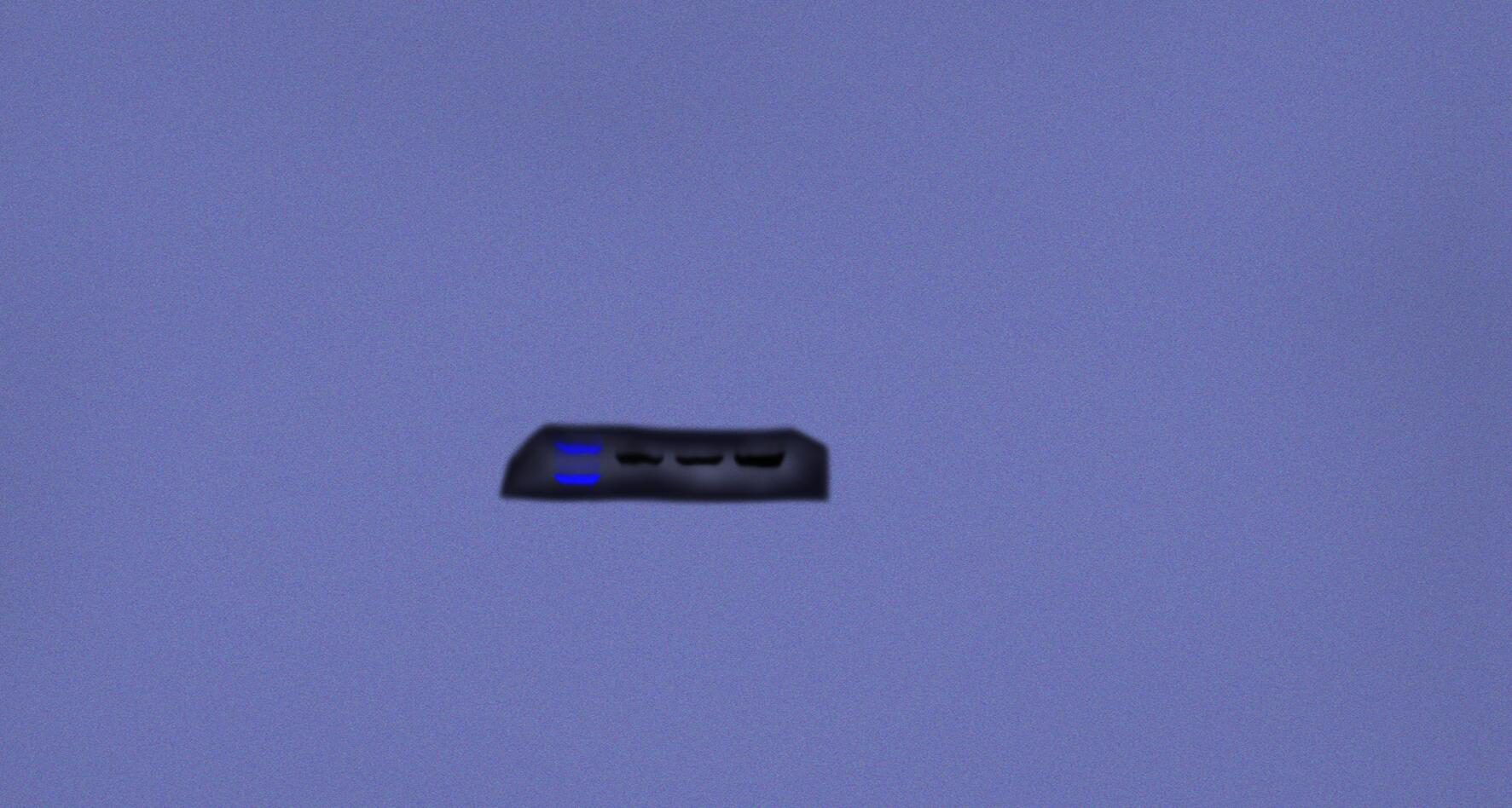

Supplement: Supplementary file 2 — (ZIP 6838 KB). [file 12672_2022_501_MOESM2_ESM.zip › SK-MES-1-WB/3-E-cadherin.tif]

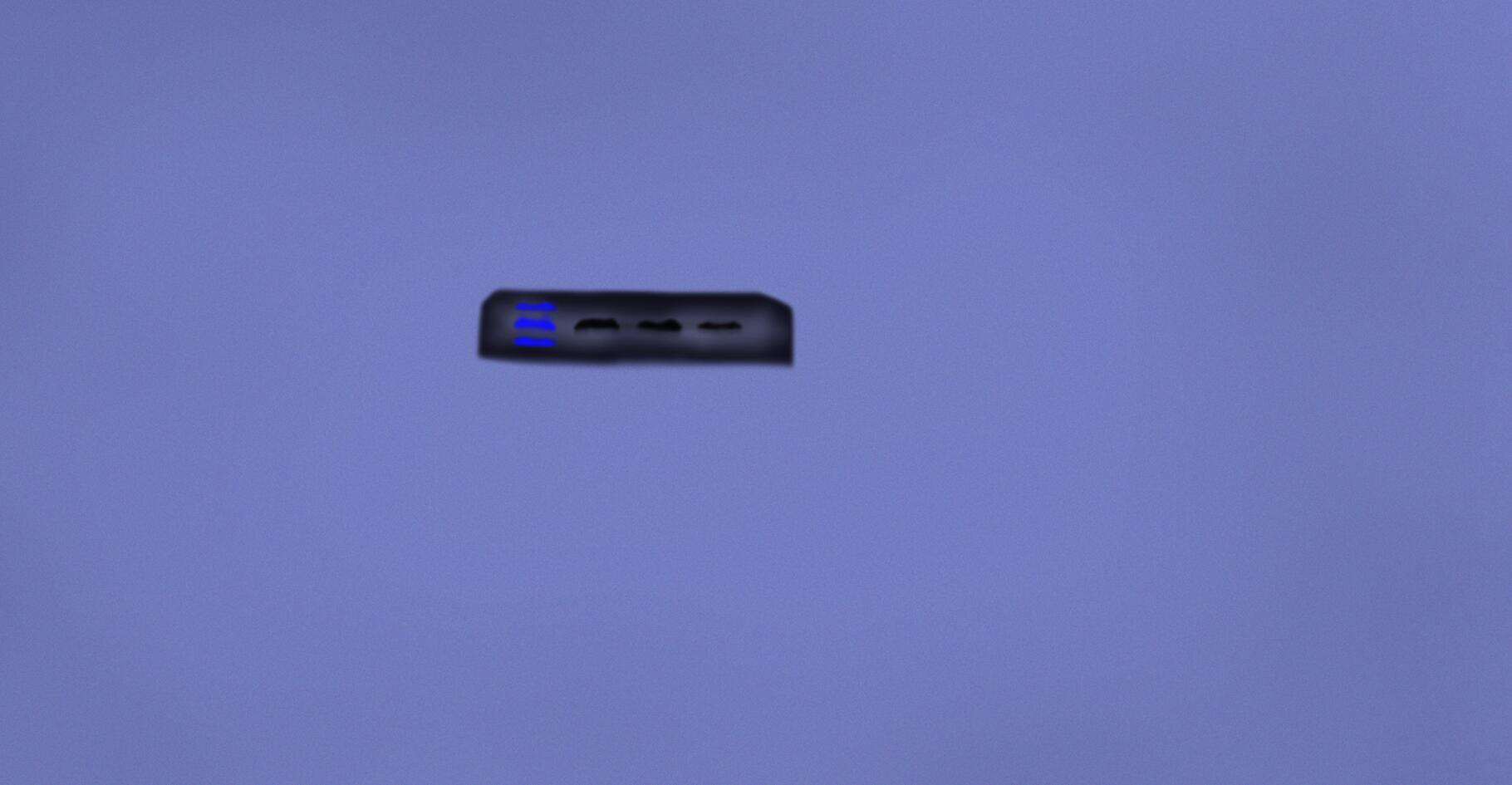

Supplement: Supplementary file 2 — (ZIP 6838 KB). [file 12672_2022_501_MOESM2_ESM.zip › SK-MES-1-WB/4-Vimentin.tif]

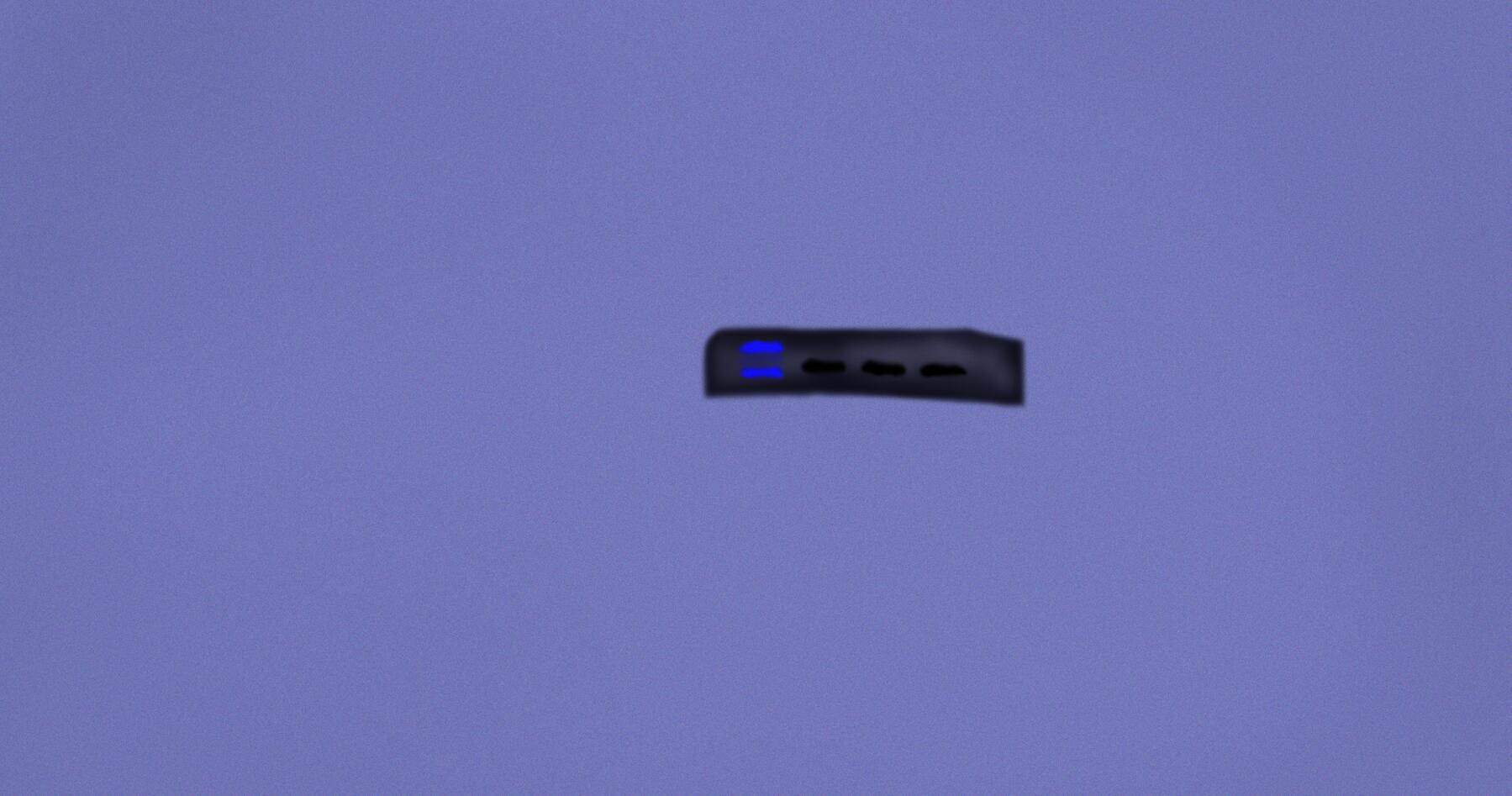

Supplement: Supplementary file 2 — (ZIP 6838 KB). [file 12672_2022_501_MOESM2_ESM.zip › SK-MES-1-WB/5-GAPDH.tif]
